# Making Markets in Long-Term Care: Or How a Market Can Work by Being Invisible

**DOI:** 10.1007/s10728-015-0292-0

**Published:** 2015-05-08

**Authors:** Kor Grit, Teun Zuiderent-Jerak

**Affiliations:** 1 Institute of Health Policy and Management, Erasmus University Rotterdam, Postbox 1738, 3000 DR Rotterdam, The Netherlands; 2 Department of Thematic Studies – Unit of Technology and Social Change, Linköping University, Linköping, Sweden

**Keywords:** Financial instruments, Health care markets, Long-term care, Market development, The Netherlands

## Abstract

Many Western countries have introduced market principles in healthcare. The newly introduced financial instrument of “care-intensity packages” in the Dutch long-term care sector fit this development since they have some characteristics of a market device. However, policy makers and care providers positioned these instruments as explicitly not belonging to the general trend of marketisation in healthcare. Using a qualitative case study approach, we study the *work* that the two providers have done to fit these instruments to their organisations and how that enables and legitimatises market development. Both providers have done various types of work that could be classified as market development, including creating accounting systems suitable for markets, redefining public values in the context of markets, and starting commercial initiatives. Paradoxically, denying the existence of markets for long-term care and thus avoiding ideological debates on the marketisation of healthcare has made the use of market devices all the more likely. Making the market invisible seems to be an operative element in making the market work. Our findings suggest that Dutch long-term care reform points to the need to study the ‘making’ rather than the ‘liberalising’ of markets and that the study of healthcare markets should not be confined to those practices that explicitly label themselves as such.

## Introduction: How to Frame ‘Marketisation’?

The academic debate on introducing ideas, principles and instruments of markets into healthcare sectors is a road full of pitfalls. What we mean by markets in the healthcare setting is a complicated question due to the slippery nature of the term ‘market’ [27, 19] and the broad ideological battle over the role of markets in healthcare. In many countries, the role of markets remains an ideological dividing line and dominant theme in health policy [38]. Some governments explicitly believe that the healthcare sector could learn something from markets or business practice. In the UK, Germany, Switzerland and the Netherlands, various governments have defended a market-oriented approach to hospital care and/or healthcare insurance to improve the efficiency, quality and responsiveness of healthcare [15, 18, 32]. Whereas at times healthcare reform initiatives are explicitly positioned in relation to ideological commitments about the role of markets in healthcare, interestingly, governments sometimes introduce highly similar instruments without relating them to the term market. By staying away from the suggestion of a market-oriented approach, governments stay out of the ideological battle even though they are still following a marketisation path. In response, it is sometimes suggested that public systems are “sleepwalking into the market” [11: 1850]. An interesting example is the introduction of care-intensity packages in Dutch long-term care, where the amount of money a care provider receives for a client is related to their individual need. These packages are not presented as market instruments and yet seem to be an attribute of markets. This example of introducing market elements without reference to the concept of markets makes an interesting case for reflecting on how we should typify ‘marketisation’. How to frame this kind of policy reform that is happening in many Western countries, especially when it is not taking place under the explicit heading of introducing market elements?

Analysts across the spectrum of opinion state that the simplistic dichotomous framework of market versus government is not fruitful for the analysis of healthcare reforms in Western countries [27]. White [38: 395] argues that “to classify policy choices as ‘market’ versus ‘government’ or ‘competitive’ versus ‘regulative’ is likely to confuse an analysis of alternatives.” These common labels deflect attention from how and why policies work and are not useful analytical terms to frame our investigation of the developments in the healthcare sector [14, 38]. Whereas terms like ‘regulated market’, ‘quasi-market’ or ‘managed competition’ [8, 9, 18] suppose that institutions are better classified on a continuum between state and markets, or that they are hybrid oxymoronic institutions that combine regulation and competition, scholars in the emerging field of social studies of markets would state that such terms are a pleonasm: one cannot think of markets *without* regulation. The introduction of market mechanisms in healthcare can be part of a more market-oriented approach, but it need not imply that this sector should be labelled a ‘market’ for these mechanisms to work. In the healthcare setting, the label refers to a diverse set of market-like instruments and arrangements, such as privately owned and managed institutions, financial incentives, competition and contracting between purchasers and providers [25]. These attributes are not the same in every market. Private organisations, for instance, can range from American stockholder-owned insurance companies to non-profit sickness funds in the German tradition and not all attributes exist in every market—see the internal market in the UK with competition between publicly-owned hospital trusts. Following Wittgenstein’s concept of ‘family resemblance’, we could typify the group of markets as a family, where various resemblances between different members overlap and crisscross, but it is hard to find anything common to all [39]. Defining ‘marketisation’ as an increasing level of resemblance or becoming a hybrid member of the ‘family’ of markets allows us to circumvent the dichotomous framework of markets versus government.

The introduction of market instruments into public service provision may—intentionally or unintentionally—not be presented as such a policy reform and thus may not be easy to recognise until it has reached a late stage. Diagnosing the character of such policy reform is especially difficult due to the lengthy process of introducing market devices to the public sector [15, 31]. Incremental shifts can add up to fundamental transformations [21]. Therefore, in this paper we conduct the scholarly experiment of analysing the introduction of new devices into the Dutch long-term care sector as preparing the ground for marketisation. Acknowledging the step-by-step character of policy reform, we shift the unit of analysis from the policy object to the policy process, from the *introduction of market elements* to the long *process of market development*. We thus aim to contribute a way of studying markets-in-the-making and show its consequences for empirical research.

## Studying Processes of Marketisation

To address the question of how to study current healthcare reforms, not as the introduction of clearly identified market elements but rather as processes of marketisation in public service domains, we draw upon the study of technology development in the field of Science and Technology Studies (STS) and on the study of market development in the field of Institutional Policy Science (IPS). The former has developed an extensive repertoire for studying the inherent dynamics and consequences of technology development, where it explicitly includes changes in the technology after their introduction. The latter has produced a conceptual machinery for analysing gradual institutional change that allows us to study the introduction of a market device before it is *presented* as an institutional reform. Both scholarly domains, commonly carried out in relative professional isolation, recognise that the policy instrument is not a fixed entity and that policy instruments may have unintended effects (market development can be an emergent aim rather than a clearly recognised goal from the start).

Authors in the STS field have argued that when introducing technologies into new settings, it can be a strength if objects are not too clearly defined and ‘black boxed’. De Laet and Mol [7: 225] propose that the strength of some devices is not that they are clearly fixed but that a technology is “an object that isn’t too rigorously bounded, that does not try to impose itself but tries to serve, that is adaptable, flexible and responsive—in short a fluid object”. De Laet and Mol mobilise the metaphor of fluidity of objects to explain the success of specific technologies.

STS scholars have also analysed the introduction of market instruments or devices from the perspective of performativity, which focuses on the *construction* of markets with the aid of market devices. According to Callon and Muniesa [3], markets cannot exist without a set of market devices. Through the notion of ‘device’, objects (the care-intensity package in our case study) can be considered as objects with agency: “whether they might just help (in a minimalist, instrumental version) or force (in a maximalist, deterministic version), devices do things” [25: 2]. Devices may therefore contribute to ‘discipline’ behaviour and to market-like decisions. However this discipline is not mechanical, irreversible or irrevocable. “It evolves and transforms itself since the tools, those solid points in the system, are themselves plastic, open, reconfigurable and, moreover, constantly reconfigured” [1: 26]. Despite efforts to frame a market with devices, framing can never be accomplished as any frame is incessantly subject to *overflowing,* or what economists refer to as ‘externalities’ [1]. Recognising this *bricolage* or tinkering with devices, framing work is never over, for new framings are always possible [2].

This finding bears resemblance to the study of gradual institutional change in IPS [21, 31]. Institutional change is not an abrupt wholesale transformation, but more an on-going struggle, although gradual change can add up to major historical discontinuities. Market reform is not a *big bang* operation [19]. In their analysis of the evolution of market-oriented reform of hospital care in the Netherlands, Helderman et al. [15] conclude that the only reason this policy could lead to the successful introduction of a recognisable market policy was a process of policy learning that took over two decades. Referring to policy learning, they mean the “process by which policy makers and stakeholders deliberately adjust the goals, rules, and techniques of a given policy in response to past experiences and new information” (ibid.: 189). The development of the Dutch market for hospital care shows how a series of incremental (path-dependent) changes could lead to (path-breaking) non-incremental change in policy [15, 19]. Over the decades, many technical and institutional adjustments were made, and are still being made, to prepare hospitals and insurance companies for a market-based policy reform, which culminated in the Health Insurance act (ZVW) in 2006. According to Helderman et al., this incremental approach was inspired by a long-term market development strategy (introducing competition) that was repeatedly adjusted to the political and institutional setting, meanwhile continuing to shape this setting to prepare it for a market-based policy reform. “Many of these necessary adjustments could not have been foreseen, but had to be discovered and learned by policy makers and stakeholders by trial and error” [15: 204]. Similarly, Marmor et al. [22] warn politicians against an over eagerness to embrace and import policy models without properly assessing how these ideas and models work in other practices and how they may require *adjustment* rather than *implementation*. Maarse and Paulus [20] therefore state that Dutch market reform is far from finished and, since 2006, there has been a constant need for adjustment and political compromise.

Both studies, technology development in STS and gradual institutional change in IPS, point to the importance of including dynamism and process analysis when studying innovation or reform. While the scholarly domains conceptualise the process differently, both notions of fluidity and gradual institutional change provide a method of studying marketisation that matches the shift in IPS from government to governance [23], and where policy is not limited to what formal policy institutions do but is the outcome of the actions of a plethora of actors, including market devices.

However, empirical studies of gradual institutional change usually make retrospective claims, once the reform has succeeded (or not). This is unfortunate, since it presents the study of policy reform with the post hoc problem. In this paper we explore the value of a processual approach that is not ex-post, but studies policy reform before it can surely be identified as such. Sieber [30: 204] describes this challenging task as “the effort to anticipate the hitherto unanticipated, and perhaps none is so foolhardy.” Sieber followed the Mertonian approach that interventions can produce the opposite of the effect desired by their architects, and that we need, or should at least try to confront the surprising or unexpected at an earlier stage [16]. Our idea that the new financial device may render the sector susceptible to future marketisation, even if it is not presented as a marketisation device, rests on the argument that there may be some Mertonian logic going on here.

## Making Markets Instead of Liberalising Markets

We analyse the introduction of a new financial regime that may prove to be the early stages of a market-based policy change in the provision of long-term care in the Netherlands. We follow the introduction of a new ‘market-like’ device for financing long-term care: the ‘care-intensity package’, an individual-trailing budget for long-term care clients. This is interesting because this is a concrete financial instrument that bares a resemblance to a market device even if it is not presented as a kind of market mechanism. Rather, it is presented as an instrument to implement the policy aim of strengthening client-oriented care. Despite this label, we explore the value of analysing the introduction of these devices as part of a long-term process of making markets in long-term care.

Although the central government forces providers to adopt the new financial regime, providers still have and need some latitude in how to translate the new device to their local setting. Therefore we focus on the reception part of the introduction of the policy instrument [4], that is, how external devices are integrated into local policies and adapted to a particular context [26]. “The transferred elements are transformed to blend with the new context” [4: 454] while these elements also shape the receivers themselves. The meaning and impact of introducing care-intensity packages should first be studied at the coalface, where people have to deal with the device and where its meaning is largely defined. The next questions are, what influence, if any, do these practical experiences have on the policy discourse on long-term care and do they help move this sector towards a more market-driven logic.

Streeck and Thelen [31: 33] suggest that liberalisation in capitalism (a general opening-up of social and economic arrangements to the logic of ‘free’ competitive markets) “may be achievable by default: by letting things happen that are happening anyway. All that may be needed for liberalisation to progress in this case would be to give people a market alternative to an existing system based on collective solidarity, and then give free rein to the private insurance companies and their sales forces.” This view shows that Adam Smith’s famous metaphor of the ‘invisible hand’ is still informing the debate about markets. Foucault [10] criticises this laissez-faire view of the market economy as ‘naive naturalism’ that interprets market exchange or competition as something produced spontaneously. Our approach follows Foucault’s suggestion that market behaviour is not the outcome of a policy of laissez-faire. This perspective on the institutional transformation of public into market sectors conceptualises the healthcare sector as an example of making markets rather than liberalising public sectors. This approach allow us to analyse how the new *market device* of the care-intensity package and the *work* done by providers to transform these devices to their practice serves the creation of markets for long-term care.

## Methods

To analyse markets-in-the-making, we addressed two questions. First, what is the *intended* result of introducing care-intensity packages in the Dutch long-term care sector? We analysed the supposed characteristics of the device and the extent of its appearance as a financial market device. Second, what visible and invisible *work* must the providers do to adapt and transform care packages for their organisation? We focused on unintended consequences, especially if this work can be diagnosed as making markets or preparing the way for the eventual marketisation of the sector.

Studying the unintended results, the work needed to make markets work, cannot be done overnight. The development, introduction and shaping of healthcare markets takes place over extended periods marked by ubiquitous reconfigurations of organisations, market mechanisms, professionals and policy makers. Therefore, we focused our empirical analysis on two long-term care institutions, an organisation for mentally disabled people and a nursing home that we have studied since 2005. Our analysis draws upon qualitative data collected from 2005 to 2010 in semi-structured, in-depth interviews (n = 22) and focus groups (n = 2).

In 2005, we focused on new developments such as market-like financing, commercial initiatives and demand-oriented care (see [12]). At the time we collected data through semi-structured, in-depth interviews (n = 13) and focus groups (n = 2) with executives, managers, professionals and client representatives. Contrary to our initial expectations, the interviews gave the impression that the effects of the developing healthcare market are found primarily on the shop floor. In the previous study, the organisations were on the eve of introducing care packages and, some of them, commercial activities as well. We returned to these organisations to see if their first ideas on how to deal with care-intensity packages had become reality. The organisations had tried to respond proactively to the introduction of care-intensity packages. Their stance on these new financial instruments—critical yet not complete rejection—allows us to analyse how organisations adapt new policy instruments into their practice. In the organisation for disabled people, we focused on how they dealt with the new policy device of care-intensity packages. Here we interviewed (n = 6) the project manager for care packages (ZZP), one cluster manager, two location managers, one senior finance adviser and one marketing manager. In the elderly care organisation with serious ambitions to start commercial activities, we wanted to know if they had developed the commercial activities and how they related to the care-intensity packages. Here, we interviewed (n = 3) the executive, the medical manager and one care adviser. In both organisations, we interviewed people in different organisation levels, including the shop floor, to realise data triangulation.

All interviews were transcribed verbatim and analysed to explore the consequences of market mechanisms introduced some years ago and to see how these market devices had developed since then. This combination of data sources over a longer period of time allowed us to address the issues raised above. As is generally the case in qualitative research, representativeness is not the aim of the study [5]. Rather, this research focuses on a in-depth analysis of a specific case to produce precise findings that may function like a ‘golden event’ [17] in the sense that they can produce, through their specificity, interesting insights into practices that would not be captured by more general analyses of larger numbers of cases [37].

We analysed and ordered the data with the aid of themes to discover and clarify possible consequences of introducing care packages. The themes included administration and management activities, attention for business processes, how care packages are discussed with clients, private initiatives in the care organisation, and distribution issues. Both authors discussed selected data until consensus was reached on data interpretation. Both research reports were sent to the involved organisations for member checks.

The board and respondents from the care organisations gave permission for the interviews. Within Dutch jurisdiction, there was no need for ethical approval for the empirical research. To respect the privacy of the participants in this study, all data have been made anonymous.

## Explicit Policy Aims of Introducing Care-Intensity Packages

The General Exceptional Medical Expenses Act (AWBZ) is a population-wide social insurance scheme for long-term care that cannot be covered with insurance. Since 1968, it has provided security for people who are disabled, chronically ill or suffer from a mental disorder. The AWBZ regulation aimed to provide funding for long-term care, shifting the financial burden from families and charity to public funding. It kept the legally independent status of private institutions in place, except that providers of residential care were not allowed to have a profit motive. They benefited from the new funding stream, greatly expanding their capacity in the 1970s and 1980s. In comparison to other European countries, the Netherlands has a high rate of residential or institutionalised care [24].

Residential care is paid mainly from the AWBZ. Residents face fairly high co-payments, especially for the cost of living. However, they often do not pay the full charge, since their contribution depends on indication, household composition, income and, since 2014, property. Only a small minority of people who need residential care make use of commercial services outside the AWBZ. For persons to qualify for care under the AWBZ, they apply for a needs assessment decided by an independent organisation responsible for determining care needs [29]. This assessment, translated nowadays into a care-intensity package, aims at guaranteeing both that individual needs are met, and that the value of accessibility is ensured. Insurers, or the healthcare offices they designate, enter into contracts with institutionalised providers. On the basis of their care-intensity package, individual users could choose their own providers from the list of contracted care institutions.

This regulatory arrangement is based on the principle that money should follow the patient or client. It could be seen as an explicit attempt to create a market for long-term care, but is not presented as such, with the argument that there is no real choice due to the overall shortage of capacity, with substantial waiting times at many care institutions. De facto this means that provider choice is highly determined by availability. Furthermore, the fact that the largest insurance company in a region gets a 4-year government concession to carry out the purchasing role for all local insurance companies adds to the fact that long-term care is not seen as a market.

Since 1999, the Dutch Ministry of Health (MoH) has been trying to develop a system of entitlements and payments for long-term care that is not based on the average client receiving care from a care provider, but is attuned to the individual needs of each client [33, 34]. This attempt to provide more client-centred care implies that ‘products’ delivered by providers are given a ‘price tag’. It was generally understood that the old supply-oriented long-term care system was no longer equipped to serve today’s public. The proceedings of the meeting of the House of Representatives of the Netherlands state:

The public is emancipated and has made clear that it wants to find meaning in life for itself and be responsible for doing so. Anticipating this societal development is the biggest problem when it comes to modernising the AWBZ. It requires redesigning the system so that it complies with the public’s demands for more freedom of choice, more options, more of a say and more participation [34: 1].

Intending to enhance both customer choice and voice, the MoH wanted to develop new financial rules for the AWBZ. According to the ministry, flexible entitlements and thus individual-linked payments are needed to achieve tailor-made care. Entitlements should be described in specific terms so that clients can determine whether they are getting ‘value for money’ [33: 24].

In 2007, after long preparation, the MoH introduced the care-intensity packages [35]. Individual ‘indications’ (assessments) and budgets were designed to provide patients with greater choice and control over their support arrangements. The new financial framework was based on the idea of personal budgets or cash-for-care schemes,^1^ however, they are not the same. With the personal budget, patients can buy and organise their own care, including from non-professionals, for instance neighbours, friends and family, whereas the care-intensity packages are still provided in a framework of provision in kind. Introducing individual-trailing or ‘client-linked’ budgets has had consequences for providers whose payment is now output-based and who are thus actively encouraged to attract and keep clients, since clients have more exit options. The idea is that if clients are better informed about their rights or their budget (because they have a stronger position vis-à-vis their provider), they will have a better chance of satisfying their own needs and wishes. For instance, clients can choose between a 30 min bath and two 15 min showers, according to the official user guide [28]. Care packages were introduced in the AWBZ on 1 January 2009.

**Fig. 1:**
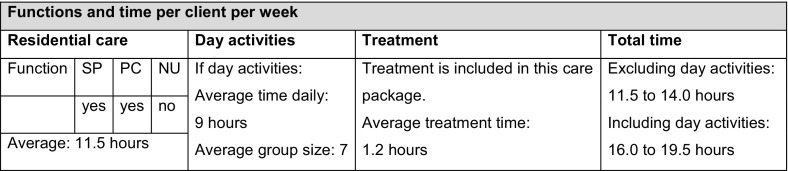
Example of a care-intensity package (category 3) for the mentally challenged

In the new scheme, the assessment is tailored to the individual client and used as a basis for the care package. For 2010, 52 care-intensity packages were defined for the three sectors of intramural care: ten for nursing homes, 13 for people with psychiatric problems and 29 for people with a mental disability. Entitlements are broadly defined. A care package, including a global indication of hours per week, describes which functions it delivers, such as support (SP), personal care (PC), nursing (NU), daytime activities and treatment. A draft version of the care package method defined entitlements more strictly, specifying the exact hours per function. Importantly, the new system permits substitution of activities. In the example shown in Fig. 1, the client can substitute support for personal care.

With client-linked or individual-trailing funding, the budget follows the client. In spite of the limited choice options for clients due to provider waiting times, the MoH propagates the idea that the output finance system will drive providers to improve the quality of care and become more client-oriented:

I expect individual-trailing funding for care in kind to act as an incentive for care providers to provide effective, good quality care in the form of an arrangement that meets clients’ preferences, since the funding is not based on the institution but on clients with particular care needs [36: 12].

Even given the providers’ lack of capacity, and if competing for clients was not a realistic option, the financial incentive could stem from clients’ awareness of their entitlements and subsequent claims to get what their indications said they were due. However, in publications on this topic, the ministry states that care packages should not lead to a fully individualised care model that allows each and every client to demand their ‘rights’. This is not in the spirit of the public AWBZ, which often offers care on a collective or group basis [36]. Thus, even though care packages were developed partly to empower the user of care, the ministry does not want the packages to lead to calculating, claim-happy care consumers. However, care-intensity packages have some characteristics of a market device, such as different prices for different clients, an output finance system, more incentives for providers and more customer choice and voice. The basic idea is that tailor-made finance should lead to tailor-made care more easily than the old funding system did.

## What Happens in Practice?

Despite the new care-intensity packages, according to many of our respondents, especially the residential part of the long-term care sector is, as mentioned above, not a real market since there is hardly any competition, insufficient choice, more demand than supply (waiting lists), and providers often have no latitude to negotiate on prices. A main reason for the scarcity of care, or waiting lists, is that the MoH maintained production quota for the AWBZ regions to protect the overall macro budget for healthcare. But even though the provider has little room to negotiate on price and volume with the insurer that has regional purchasing responsibilities, the introduction of ‘price tags’ (the budget of a care package) leads to changes in the internal organisation of the provider and in the provider-client relationship.

### Organising Care with the Aid of Care Packages

Individual care funding was driven by the idea that clients are good at determining whether they get value for money. As a middle manager of an organisation for the mentally disabled said: “There is more pressure on accountability, so we have to look at how we can make things clearer”. With ‘things’ he referred to the care and services clients were receiving. However, providers also said that they need a workable form of accountability. Complete transparency of individual care package spending requires extensive registration of total hours or activities. Collective accountability at location level is easier for providers, especially in situations where clients live in groups and receive collective care. Moreover, the accountability to care offices and clients is easier for groups with the same type of clients and care-intensity packages. To create a workable situation, the provider does not strive for complete transparency of care package spending per client, except for some information on duration, including collective or group hours. This transformation of individual care packages into collective arrangements also creates, according to one manager, some latitude for caregivers to trade clients’ individual fluctuations in daily needs (cluster manager, organisation for disabled people).

Respondents indicate that the individual funding system has increased insight into business processes and providers are better equipped to benchmark locations internally and instigate improvements. They can compare locations in terms of economic performance and whether locations are faced with over- or underproduction issues.

If you saw underproduction and overspending in one location, well then you’d have to deal with a reinforcing effect. Then you’d call that location to account one day, depending on the degree of underproduction and overspending (senior financial adviser, organisation for disabled people).

Using the care-intensity package tool, managers can see how their location performs compared to other locations in the same cluster. The improved transparency of the results at location level is driving providers to adjust supply better to the indication. Responsibility for budgetary control has shifted partly from the organisational level down to lower levels. To balance their books, organisations used to shuffle funds between their various budgets (substitution). Given the fact that one tariff was used for all clients, departments that had not spent their entire budget could compensate for departments that overspent their allocations. Nowadays, this form of substitution has become less acceptable than it was in the past (location manager, organisation for disabled people).

Another consequence of the new financial structure is the clustering of similar types of clients. For instance, in the past, different degrees of disability were placed in one group. In the present system, providers want to stop combining ‘lighter’ and ‘heavier’ clients, as the professional level of caregivers has to be attuned to the more severe client type (middle manager, organisation for the disabled). The increased transparency generated by the new financial structure stimulates the provider to cluster the same type of clients to save on overhead and staffing costs. The provider no longer wants to rule out the possibility of moving clients to another location because of related efficiency gains.

Not all these consequences are directly caused by the individual funding system. According to one respondent, lower budgets for care packages force the organisation to develop efficient ways of providing care or take more notice of the business process (senior financial adviser, organisation for disabled people). In any event, care packages help providers to make clear which of their locations are inefficient or not remunerative enough and where they need to change their organisation of care. Providers are answering questions on location performance increasingly by using an internal market perspective of profitability of locations. Although budget reductions partly induce this, the financial instruments are instrumental in this development.

### Care-Intensity Packages as a Tool to Substantiate the Possible

Since providers are not searching for standardisation in terms of concrete activities, hours or Euros, care-intensity packages are used only to describe in general what the client can and cannot expect from the provider. Respondents mention that care-intensity packages have the advantage of making clearer what a provider will or will not deliver. They can use the care-intensity package as a tool to start talking with clients or to check if they have really put their money where their mouth is (actually achieve what they advocate). As in a regular market, the provider can now delineate the possible and substantiate what is not possible:

The old system just could not do that. Then it was just indications for residence and care. Okay, so now you get emancipated clients who think that every normal request is possible. And now you can make clear that a lot is possible, yes, but within limits. You can only opt for something at a given moment. (…) It’s like, look, if I buy a new car that means I can’t also buy, let’s say, a new bike or audio equipment. I mean, I can only spend my euro once. You see? In that sense you can make things transparent (financial adviser, organisation for the disabled).

However, according to our respondents, client representatives almost never use the care package as an aid to talking about the amount or quality of care. They are more interested in how things are going and if the care is all right for them. Respondents experienced that clients seldom use the care package as an instrument for demand-driven care.

Interestingly, individual funding tends to strengthen the supplier’s position. As we have shown, care-intensity packages clarify which services should or should not be delivered. In other words, with the aid of these financial instruments, care can be better attuned to the providers’ possible options. It becomes easier to determine unreasonable demands and when to say “No” to clients. Under the guise of demand-orientation, clients are required to fit their demands to the care package they receive. Providers and clients have thus transformed a demand-oriented instrument into a supply-oriented instrument. If providers gain more power than clients then the policy would be counterproductive in terms of inducing client-oriented care. However, this specific use of the new financial device fits with other forms of market behaviour, where providers are accustomed to limiting their efforts to the price consumers are prepared or able to pay.

### Clarifying the Limits of Publicly Financed Care and Commercial Initiatives

Since long-term care is publicly financed by the AWBZ, many clients or their families expect the provider to deliver everything. That is not always the case. For instance, both providers explain that they simply lack the personnel to cope with residential clients who want assistance for going outside as soon as the weather is nice. Private finance creates an important option to offer more than what is possible under a budget funded by the AWBZ. However, the Dutch publicly financed system of long-term care has no tradition of paying for services, except for income-related contributions to residential care. A few years ago, the organisation for elderly care we studied wanted to develop ‘plus’ packages with extras that clients could buy [13]. The executive of that organisation said in 2007 that he wanted to develop products that could make life more pleasant for his residents:

If people say, “We want our mum or dad to have an hour’s walk outside every day” then we’d have to say sorry, that’s not in the standard care package, but we can do something about it. We’ll arrange it for you, but it means having to send you a plus package bill (executive, organisation for elderly care).

Commercial activities are seen as an attractive improvement to the healthcare system, especially because they are presented as extra options on top of the existing care-intensity packages. A few years later, in contrast to this provider’s ambitions, the supply of plus packages or commercial activities is developing very slowly. According to our respondents, there is only a limited range of products and services, such as foods and beverages, laundry service, bedside TVs and party site rentals for birthdays. Offering paid extras in the publicly funded part of the market is a complicated matter, as staff members indicate that they find it hard being allowed to offer some clients a specific service and then having to say no to other clients who cannot afford it. This does not mean that residents do not accept differences: clearly, it is common practice that some call a taxi and if family can make a difference, staff will certainly react well. Problems arise if *employees* have to make distinctions in, for instance, who gets the opportunity to go outside: “It feels different when one of the staff is paid to come in and do that [accompany a fragile elderly client outside]. It’s different from someone calling a taxi to go out” (client adviser, organisation for elderly care). Interestingly though, individual funding can be used to make the distinctions clearer between basic care and the extras that people have to pay for.

You need to make the difference clear between what you need and what else you’d like, and what to do if that little bit extra goes beyond the basic package. Now we have a good reason to explain all this. Now you can make it clear with the help of those care-intensity packages. It gives people a choice (client adviser, organisation for elderly care).

Care-intensity packages can be used to clarify the limits of publicly funded institutional care. The new financial system makes it more transparent what should be part of the publicly financed compartment and hence what can be offered by plus packages or has to be provided by family members and volunteers. Although this device was developed for the publicly funded part of long-term care, providers are trying to use it to stimulate and initiate private market initiatives and are finding out this distinction is extremely hard to maintain in practice. They are also experiencing that it is easier to use these devices to delineate the basic package rather than to offer additional services.

### Redefining Distributive Justice

With the introduction of care-intensity packages in long-term care, distributive justice could gain a new meaning. In the internal allocation of resources, the operating distributive principle of both providers gets reconfigured. People receive an indication based on need. However, when people with an indication (care package) go to the provider, the care package is transformed partly from a ‘need’ into an ‘economic demand’. Traditionally, because of similar funding budgets and different needs, providers employed financial substitution between departments, where departments with spare money (unspent budget) compensated for departments that had overspent theirs. Nowadays, every demand based on indication or care package is taken equally seriously, which means that shuffling budgets becomes increasingly inappropriate. At the start of the new financial scheme, one of the providers was concerned that substitution would no longer be possible in future [12]. If the right to care has become defined by client budgets based on care packages, any shifting of funds between budgets could increasingly be interpreted as in conflict with the rights of individuals and thus unwittingly gain the connotation of trickery, or even fraud, he feared:

You know, we used to shunt budget money around our departments and between patients. Nowadays you couldn’t get away with that. If I did try it, I wouldn’t be able to explain my expenditures to the accountant or to clients. I’d run into accounting problems (division manager, organisation for the mentally disabled).

A few years later, this idea had not entirely come true. Nowadays, this provider has chosen to keep intact some form of substitution at the level of the organisation. There still is room for substitution between locations in specific situations, although this mechanism will be used less frequently than in the past and there are providers that have completely rejected this option. In the location, care can still be distributed according to the principle of need. One respondent explained that their organisation is prepared to defend the fact that something must be deducted from the care-intensity package in order to give specific groups the care they need. He considers this a part of their societal responsibility: “We find it important that we can give extra care when someone needs extra help. (…) You can explain that story” (executive, organisation for the mentally disabled). Providers have some latitude in how they translate the individual care-intensity package into their collective care practice. Although this could be understood as showing the acting space of providers to accommodate individual funding schemes in collective care arrangements, providers are in fact contributing to the legitimacy of introducing market mechanisms that risk focussing on individual demand in collective healthcare settings. Now they may have to defend practices explicitly to clients and accountants that they could do invisibly before.

## Discussion: Studying Preliminary Market-Making Work

Recognising the slippery nature of the term ‘market’ enables us to take a broader approach to marketisation that is informed by theoretically studies on fluid technology development and gradual institutional change. This redirects the scholarly focus from ideological quarrels about healthcare markets to the study of processes of market-making. In our case, even if long-term care in the Netherlands is persistently articulated as a non-market setting, the introduction of the care-intensity packages and the way they are reconfigured in practice is leading to changes that could bring a market for such care a step closer or increase its resemblance to others in the ‘family’ of markets.

We have presented the Dutch long-term care sector as an example of a market development that does not have such an explicit policy goal. Paradoxically, denying the existence of markets for long-term care has made the use of market devices more likely. Presenting the new instruments as reinforcements to client-oriented care leaves room for providers to search for workable local solutions, such as defining the boundaries of the care-intensity package that will be crucial for the potential next steps in developing the market for long-term care. The providers have entered the process of marketisation based on the introduced market devices, although (for now) they reject the idea that the long-term care sector resembles a market in any substantial sense. Providers have done all kinds of preliminary market-making work, such as creating suitable individualised accounting systems, clarifying the limits of publicly financed care, redefining (public) values in the context of markets and starting commercial initiatives. They have done much of the work that can be classified as market development.

Our case study suggests that market development is made possible by changing rather than rigidly implementing the market device in local settings. Similarly, in opposition to the suggestion that long-term care is not suitable for market development, we find that providers can deal with the market device of care-intensity packages, as long as they try to mould the introduced market device in such a way that it leads to workable and justifiable solutions, which in turn also shape both the care providers and the clients, as well as (the limits to) their entitlements. Their work is crucial for the suitability and legitimacy of market development. Output finance requires providers to justify and make clear how they spend client-linked budgets; however complete accountability and transparency is not workable. Providers have to decide how to spend these budgets and how to apply individually client-linked budgets to group-based services. They have to decide to what extent substitution between budgets is acceptable. Justifying this substitution to clients is also done by using entitlement boundaries to make clients accept the limits of care service delivery. This shows that providers have tried to transform market devices so that they can be embedded in the Dutch tradition of collectively organised long-term care, thus creating more legitimacy for a market-making policy process that makes a market not at odds with collective concerns. At the same time, the idea of collective care arrangements is not unaffected by the market devices because of clearer limits of individual entitlements in the collective arrangements. The government’s introduction of markets devices in combination with the providers’ work of fitting these into the traditional provision of collective care shows that our case is more an example of ‘making markets’ than a case of liberalising long-term care to let ‘the market’ work. Although the policy actors involved do not embrace market discourse, when analysing this case in terms of the genealogy of policy reform, our study shows that these devices are rendering the Dutch long-term care sector susceptible to further market development and collective provision is likely to stay part of these future markets.

Following this analysis, it is no surprise that subsequent Dutch governments want to continue the process of market development. One proposed measure is to separate housing and care, which would reduce public expense while allowing it to be presented again as offering more options outside the publicly financed basic package—with professionals having to somehow make such options legitimate in collective arrangements. Other measures are a complete cutback of funding based on the institution to strengthen output finance and shifting the purchasing task to individual insurers, which should stimulate insurers to focus more on their clients. These proposals are not new, but the introduction of care-intensity packages has created a market framing and an accounting infrastructure which puts them back onto the policy agenda and renders them increasingly easy to imagine. With the help of our market-making approach, we can conclude that care-intensity packages have prepared the sector for these new proposals, although this was not their aim. Since 2015, carrying out the AWBZ is no longer a task for the central government: it has been delegated to three actors: light forms of care and mental healthcare are delegated to private insurers, 24–7, heavy care is carried out by the largest regional insurer with a purchasing concession, and youth care has been transferred to the municipalities. This transfer of AWBZ entitlements to the domain of the already market-oriented Health Insurance act implies further steps towards market development that would have been hard to imagine without earlier market-making work. At the same time, it would have been equally hard to imagine how these market instruments for individualising entitlements could become part of a collective understanding of long-term care provision, albeit a collective that is increasingly safeguarded by applying clearer limits on entitlements to say ‘no’ to clients. Our analysis shows that it is not clear if clients are more satisfied now, following the introduction of care-intensity packages. We recommend more research into the experience of care users in the introduction of marketisation policies, especially when these reforms are justified with reference to client demands.

Inspired by studies of technology innovation and market development, we propose a policy reform approach that stresses the greater importance of fluidity above a rigidly clear definition of policy. Market development is not just a technique of fitting a market device into a different jurisdiction or environment, but a dynamic process of reciprocal adaptation. This surely points to the need to study the ‘making’ rather than the ‘liberalising’ of markets. And this ‘making’ can start well before labelling initiatives in terms of markets. Therefore, the study of markets in healthcare should by no means be confined to those practices that explicitly label themselves as such.

## Footnotes

1 See Da Roit and Le Bihan [6] for an overview of these schemes in six European countries.
